# Cardiac Performance of Free-Swimming Wild Sockeye Salmon during the Reproductive Period

**DOI:** 10.1093/iob/obz031

**Published:** 2019-12-18

**Authors:** T S Prystay, R de Bruijn, K S Peiman, S G Hinch, D A Patterson, A P Farrell, E J Eliason, S J Cooke

**Affiliations:** 1 Fish Ecology and Conservation Physiology Laboratory, Department of Biology and Institute of Environmental Science, Carleton University, Ottawa, Canada; 2 Department of Forest and Conservation Sciences, University of British Columbia, Vancouver, Canada; 3 Department of Zoology, University of British Columbia, Vancouver, Canada; 4 Fisheries and Oceans Canada, Cooperative Resource Management Institute, School of Resource and Environmental Management, Simon Fraser University, Burnaby, Canada; 5 Department of Ecology, Evolution, and Marine Biology, University of California Santa Barbara, Santa Barbara, CA, USA

## Abstract

Researchers have surmised that the ability to obtain dominance during reproduction is related to an individual’s ability to better sequester the energy required for reproductive behaviors and develop secondary sexual characteristics, presumably through enhanced physiological performance. However, studies testing this idea are limited. Using sockeye salmon (*Oncorhynchus nerka*), we explored the relationship between heart rate and dominance behavior during spawning. We predicted that an individual’s reproductive status and energy requirements associated with dominance can be assessed by relating routine heart rate to changes in spawning status over time (i.e., shifts among aggregation, subordinance, and dominance). Thus, we used routine heart rate as a proxy of relative energy expenditure. Heart rate increased with temperature, as expected, and was higher during the day than at night, a known diel pattern that became less pronounced as the spawning period progressed. Routine heart rate did not differ between sexes and average heart rate of the population did not differ among reproductive behaviors. At the individual level, heart rate did not change as behavior shifted from one state to another (e.g., dominance versus aggregation). No other trends existed between routine heart rate and sex, secondary sexual characteristics, survival duration or spawning success (for females only). Therefore, while our study revealed the complexity of the relationships between cardiac performance and reproductive behaviors in wild fish and demonstrated the importance of considering environmental factors when exploring individual heart rate, we found no support for heart rate being related to specific spawning behavioral status or secondary sexual characteristics.

## Synopsis

Rendimiento Cardíaco del Salmón Rojo Salvaje que Natación Libre durante el Período ReproductivoLos investigadores han conjeturado que la capacidad de obtener dominio durante la reproducción está relacionada con la capacidad de un individuo de obtener mejor la energía requerida para los comportamientos reproductivos y desarrollar características sexuales secundarias, presumiblemente a través de un mejor rendimiento fisiológico. Sin embargo, los estudios que prueban esta idea son limitados. Usando Salmón rojo (*Oncorhynchus nerka*), exploramos la relación entre la frecuencia cardíaca y el comportamiento de dominación durante el desove. Predijimos que el estado reproductivo de un individuo y los requisitos de energía asociados con el dominio pueden evaluarse relacionando la frecuencia cardíaca de rutina con los cambios en el estado de desove a lo largo del tiempo (es decir, cambios entre la agregación, la subordinación y el dominio). Por lo tanto, utilizamos la frecuencia cardíaca de rutina como un proxy del gasto energético relativo. La frecuencia cardíaca aumentó con la temperatura, como se esperaba, y fue más alta durante el día que por la noche, un patrón conocido que se hizo menos pronunciado a medida que avanzaba el período de desove. La frecuencia cardíaca de rutina no difirió entre sexos y la frecuencia cardíaca promedio de la población no difirió entre los comportamientos reproductivos. A nivel individual, la frecuencia cardíaca no cambió a medida que el comportamiento cambió de un estado a otro (i.e., dominación vs. agregación). No se observaron otras tendencias entre la frecuencia cardíaca y el sexo de rutina, las características sexuales secundarias, la duración de la supervivencia o el éxito del desove (solo para hembras). Por lo tanto, si bien nuestro estudio reveló la complejidad de las relaciones entre el rendimiento cardíaco y los comportamientos reproductivos en el Salmón rojo y demostró la importancia de considerar los factores ambientales al explorar la frecuencia cardíaca individual, no encontramos respaldo para la frecuencia cardíaca relacionada con el estado conductual de desove específico o características sexuales secundarias.Translated to Spanish by J. Heras (herasj01@gmail.com)

Desempenho Cardíaco de Natação Livre em Salmão Sockeye Selvagem durante o Período ReprodutivoOs pesquisadores supuseram que a capacidade de obter dominância durante a reprodução está relacionada à capacidade de um indivíduo em melhor obter e aplicar a energia necessária para comportamentos reprodutivos e desenvolver características sexuais secundárias, presumivelmente por meio de um desempenho fisiológico aprimorado. No entanto, os estudos que testam essa ideia são limitados. Usando salmão sockeye (*Oncorhynchus nerka*), exploramos a relação entre a frequência cardíaca e o comportamento de dominância durante a desova.Previmos que o estado reprodutivo de um indivíduo e os requisitos de energia associados à dominância podem ser avaliados relacionando a freqüência cardíaca de rotina às mudanças no status de desova ao longo do tempo (ou seja, mudanças entre agregação, subordinação e dominância). Assim, usamos a frequência cardíaca de rotina como um indicador do relativo gasto energético. A frequência cardíaca aumentou com a temperatura, conforme o esperado, e foi maior durante o dia do que à noite, um conhecido padrão de diel que se tornou menos pronunciado à medida que o período de desova progredia. A frequência cardíaca de rotina não diferiu entre os sexos e a frequência cardíaca média da população não diferiu entre os comportamentos reprodutivos. No nível individual, a frequência cardíaca não mudou à medida que o comportamento mudou de um estado para outro (por exemplo, dominância versus agregação). Não foram observadas outras tendências entre freqüência cardíaca de rotina e sexo, características sexuais secundárias, duração da sobrevivência ou sucesso da desova (somente para mulheres). Portanto, embora nosso estudo tenha revelado a complexidade das relações entre desempenho cardíaco e comportamentos reprodutivos em peixes selvagens e demonstrado a importância de considerar fatores ambientais ao explorar a frequência cardíaca individual, não encontramos suporte que frequência cardíaca esteja relacionada ao status comportamental de desova específico ou à características sexuais secundárias.Translated to Portuguese by Diego Vaz (dbistonvaz@vims.edu)

## Introduction

Dominance is a common strategy used to enhance reproductive success (see Ellis 1995). Dominance occurs when an individual has a characteristic or a resource that provides greater reproduction opportunities than other individuals, resulting in asymmetric fecundity among individuals in a population (Huntingford and Turner 1987; Reeve et al. 1998). It has been suggested that attaining such characteristics or resources is related to an individual’s physiological performance (see De Gaudemar and Beall 1999; Creel 2001; Sloman and Armstrong 2002; Perry et al. 2004). Yet, most research conducted on fish, mammals, and birds has focused on relating reproductive behavior with morphological variables such as size (e.g., Haley et al. 1994; Quinn and Foote 1994; Tentelier et al. 2016) and secondary sexual characteristics (Shine 1979; Creel 2001; Clutton-Brock 2009), or long-acting sex hormones (Brantley et al. 1993; Oliveira et al. 2001; Kuerthy et al. 2016), rather than focusing on physiological capabilities. Therefore, research assessing the role of real-time variation in physiological performance on dominance behavior during reproduction is limited. This knowledge gap reflects, in part, the inherent challenge of assessing physiology in free-living animals in real-time (Costa and Sinervo 2004; Spicer and Gaston 2009). Nonetheless, an increasing number of electronic tagging tools are beginning to enable researchers to do so (reviewed in Cooke et al. 2004). Moreover, physiology (e.g., an individual’s capacity for exercise such as aerobic scope) and behavior are inherently linked and are important considerations for a mechanistic understanding of ecological processes and individual fitness (Gilmour et al. 2005; Cooke et al. 2014; Killen et al. 2013). Assessing the relationships between the variation in intraspecific physiological performance and reproductive fitness improves our understanding of natural selection on physiological mechanisms associated with an individual’s reproductive behavior, such as dominance and subordinance, or determining when an animal is ready to start competing with others. Consequently, exploring such linkages associated with reproductive behavior, as we do here, is particularly important for understanding the evolutionary ecology of organisms, and how this relates to changing environments (McNamara and Houston 1996; Pörtner and Farrell 2008).

To explore how physiological performance relates to reproductive behavior, Pacific salmon (*Oncorhynchus* spp.) are a particularly useful model. Being semelparous, they have one opportunity to generate lifetime reproductive success because they die after spawning, and being anadromous, they cease feeding in freshwater while migrating and spawning and therefore rely on internal energy stores to fuel all reproductive activities. Additionally, Pacific salmon spawn in high densities, where they compete for territory and mates (Healey et al. 2003; Esteve 2005; Quinn 2005). For example, males compete with each other to mate with several females, while females compete with each other for high-quality redd (i.e., nest) sites and males (Quinn 2005). In fact, both male and female Pacific salmon spawn with on average 2.5 partners (Mehranvar et al. 2004). Competitive behaviors include *charging*, where a fish swims up to a conspecific and, in some cases, bites or rams it, and *chasing*, where one fish continues to charge a retreating conspecific (Healey et al. 2003; Esteve 2005). Males also use *posture* displays, where the nose is pointed upward and the dorsal fin is erect, and *lateral displays* where the body is tensed and fins are spread as a possible warning to other approaching males (describe by Healey et al. 2003). As a female approaches oviposition, the dominant male must deter the subordinate males (satellite and sneaker males) that become increasingly active (Esteve 2005). After a spawning event, a female either builds another redd to spawn again or defends her first redd. Males then assume a subordinate role, but continue to compete for nesting females, either as a subordinate or by regaining dominant status. Hence, subordinate males and females are those that have either already spawned or have yet to spawn, but do not have a redd, and are trying to dominate over another individual, or are attempting to sneak a spawning opportunity from a dominant individual (males only). Thus, while dominance is important for reproductive success, an individual’s dominance status can vary considerably during the spawning period (De Gaudemar and Beall 1999; Mehranvar et al. 2004).

An individual’s energy state is expected to affect their physiological condition and therefore reproductive success (Alonzo and Warner 2000; Nadeau et al. 2010; Brownscombe et al. 2017). Sockeye salmon (*O. nerka*) consume about half of their total stored energy after entering freshwater to spawn (Brett 1983; Hendry and Berg 1999; Crossin et al. 2004). Energetically costly spawning behaviors related to dominance likely include aggression (i.e., charging, chasing, posture display), redd construction, courtship, and quivering (high frequency, full body shakes) (Webb and Hawkins 1989). Furthermore, electromyogram (EMG) telemetry suggests that digging is the most energy demanding behavior for females, while posture display is the most instantaneous energy demanding behavior for males. However, prolonged holding (remaining in one spot; performed by both spawners and non-spawners) generated the greatest overall energy demand by being the most common behavior by far (Healey et al. 2003). The prediction that energy-demanding spawning behaviors will require higher physiological performance seems likely because spawners expend roughly three times more energy per day than non-spawners (Healey et al. 2003). However, a direct assessment of this relationship has yet to be made.

The cardiovascular system is a logical biological unit to assess physiological performance given that it is responsible for distributing oxygen, nutrients, hormones, and cellular waste (reviewed in Hoar et al. 1992; Pörtner and Farrell 2008). However, cardiac output has yet to be measured in free-swimming fishes. Instead, heart rate (*f*_H_) is being used as a proxy because of a strong relationship between metabolic oxygen consumption and *f*_H_ under certain situations (Clark et al. 2010; Eliason et al. 2013). For example, variation in *f*_H_ has been successfully used in salmonid studies to assess the relative physiological effects of temperature (Steinhausen et al. 2008; Eliason et al. 2011), fisheries interactions (Raby et al. 2015; Prystay et al. 2017), and feeding (Eliason et al. 2008; reviewed in Farrell et al. 2009). Furthermore, Eliason et al. (2013) demonstrated that energy acquisition and tolerance to stress is limited by the cardiovascular system in salmonids. Nevertheless, changes in *f*_H_ can only be a relative measure of energy expenditure because exercising fish can alter oxygen delivery by regulating cardiac stroke volume and tissue oxygen extraction independent of *f*_H_ (Farrell, 1984). Therefore, the present study tested the prediction that *f*_H_ is related to spawning and reproductive behaviors using individual, wild, free-swimming fish for the first time. We used *f*_H_ biologgers to explore the relationship between the scope for *f*_H_ in sockeye salmon in a spawning channel while monitoring their reproductive status (dominant, subordinate, aggregation), longevity in the spawning channel, and spawning success (females only).

## Methods

### Data collection

This study took place at the Gates Creek spawning channel, D’Arcy, British Columbia, on the N’aquatqua First Nations land (50.5481°N, 122.4832°W). The spawning channel is a man-made channel and consists of a closed system that is narrow (8 m wide), shallow (∼0.5 m deep) with deeper interspersed pools, and just under 2 km long permitting individual fish to be easily identified and monitored. In 2016, from August 24 to September 12, a total of 64 sockeye salmon were individually dip netted from the entrance to the spawning channel. During the transfer, fish were electro-sedated using fish handling gloves (Smith-Root, Inc., Washington, DC, http://www.smith-root.com; 25 mA) and maintained in a trough with flowing water from the channel while a *f*_H_ bio-logger (DST milli-HRT, 13 mm × 39.5 mm, Star-Oddi, Iceland; http://www.star-oddi.com/) was implanted as described in Prystay et al. (2017). Bio-loggers recorded *f*_H_ every 5 min at 100 Hz and a raw electrocardiogram (ECG) every 1.5 h to validate signal quality. A 2 mL caudal blood sample was collected (a heparinized vacutainer with a 21-G needle and lithium heparin; BD, Franklin Lakes, NJ) to measure hematocrit using heparinized capillary tubes (75 mm Drummond Hemato-Clad, ammonium heparin) centrifuged at 8000 *g* for 5 min.

Secondary sexual characteristics were then assessed by measuring (mm) fork length, vertical depth measured immediately before the dorsal fin, kype length (distance from eye to nose), and circumference immediately before the dorsal fin, as well as a qualitative score for color (0 = silver; 1 = dark silver with some red; 2 = light red; and 3 = red). Fish condition was assessed using a qualitative injury score (0 = no injuries or fungus; 1 = minor scratches; 2 = scratches and fungus; and 3 = portion of tissue missing and fungus; a similar system has been used in previous studies, e.g., Raby et al. 2015) and the percentage body fat from a handheld microwave meter (Distell Fish Fatmeter FM 962, Distell.com Inc., West Lothian, UK). Individual fish were visually marked by placing a spaghetti tag and a uniquely numbered (color coded for sex) Peterson disk tag on either side of the dorsal musculature near the dorsal fin. Finally, the wet mass (g) was obtained by placing the fish in a rubber holding bag suspended from a spring-loaded scale. At this point, the fish were no longer sedated and were immediately released into the channel where they swam into low-flow water.

Starting the day after release, individual sockeye salmon behavior in the spawning channel was monitored daily. Fish were identified from the channel banks using binoculars, while ensuring not to disturb the fish. Behavior assessments lasted roughly 2 min, enough to assign each individual to one of three observable behaviors: aggregating, dominant, or subordinate (Healey et al. 2003; Esteve 2005). Aggregation was defined as when a fish was clustered with conspecifics in holding pools. Dominance was defined as when a fish was in a position to spawn (females were on a nest, digging and chasing other fish, while males were defending a territory and outcompeting other males when challenged). Subordinate was defined as when a fish attempted to take over another fish’s territory, sneak onto a nest with a spawning female, or simply holding alone. The behavior and the time that the behavior occurred were recorded for each observation. After natural mortality, the carcasses were collected within 24 h and the *f*_H_ loggers were removed. Depth, circumference, percentage of fat, mass (using a digital scale, Ohaus Trooper), and length were re-measured. Condition factor was calculated (*K* = 100 × (weight/length^3^)). Female gonads were removed and weighed to calculate gonadal somatic index (GSI = (gonad wet weight (g)/total fish wet weight (g))  × 100) as a metric of spawning success. Fully spawned females had a GSI = 0.

This study was conducted in accordance with the Canadian Council on Animal Care guidelines and in accordance with the standards set by Carleton University (license no. 104172).

### Data processing

When possible, *f*_H_ data were validated against their respective ECG traces using the Pattern Finder 240 software (v. 1.11.0, Star-Oddi, Iceland) to verify that the logger algorithm was accurate. Additionally, the biologgers provide a quality index (QI) for each heart rate record (0 = good and 3 = poor). Therefore, all *f*_H_ records with QI = 2 and QI = 3 were removed from the dataset. However, given the loggers were programmed to assign a default QI = 3 for *f*_H_ records >100, all *f*_H_ records between 100 and 130 beats min^−1^ (maximum recorded *f*_H_ in sockeye salmon in literature; Eliason et al. 2013) were retained.

A total of 64 fish were implanted with *f*_H_ loggers, however only 55 were used for data analysis (four had logger failure and five loggers were lost). Hematocrit levels indicated that the remaining fish were in good condition, where in all cases hematocrit was >20% (average of 36 ± 0.8% SE; Gallaugher and Farrell 1998). Depth, circumference, percentage of fat, mass, length, condition factor, and hematocrit provided measures of fish condition.

### Statistical analyses

Temperature (also recorded by the biologger) ranged between 8°C and 15°C during the study period, but not all individuals experienced the same variation in temperature. A linear mixed effect model, with individual as a random effect, revealed temperature had a positive effect on *f*_H_ (Table 1). Therefore, *f*_H_ was corrected for temperature by determining the linear regression between *f*_H_ and temperature (rounded to the nearest 0.01°C) and using residuals of the linear regression in subsequent analyses. Average routine *f*_H_ was calculated from the total *f*_H_ trace. Resting *f*_H_ was assigned to the mean of the lowest 10% *f*_H_ values. Maximum *f*_H_ was assigned to the mean of the highest 5% *f*_H_ values (which may underestimate maximum *f*_H_ but we did not want to interfere with the natural spawning behavior). Scope for *f*_H_ was the difference between maximum and resting *f*_H_. To account for any individual differences in the capacity to change *f*_H_, routine *f*_H_ was also expressed as a percentage of the scope for *f*_H_ (*R*%*f*_H_) which was calculated from ((routine *f*_H_−resting *f*_H_)/scope for *f*_H_) × 100.

**Table 1:** Statistical analyses (nine tests) comparing heart rate (*f*_H_) to fish condition, secondary sexual characteristics, time in the spawning channel, and diel patterns (day vs. night).

Model type	Response variable	Independent variables	df	*F*	*P*
Linear regression with individual as a random effect
	*f*_H_	Temperature	98,349	518.8	**<0.001**
	*f*_H_	Sex	53	1.17	0.28
	*f*_H_ residsᶧ	Diel patterns	719	48.44	**<0.001**
		Time in channel	719	6.44	**0.011**
		Diel patterns: time in channel	719	6.29	**0.012**
	*f*_H_ residsᶧ	Weight	51	0.32	0.57
		Depth	51	0.009	0.92
		Injury	51	0.080	0.78
	*f*_H_ day residsᶧ	*f*_H_ night resids	321	267.8	**<0.001**
	*f*_H_ night residsᶧ	Sex	51	0.40	0.53
		Injury	51	0.05	0.83
		Color	51	0.04	0.84
					
			**Std. error**	***Z***	***P***
					
Beta regression	*R*% *f*_H_ᶧ	Sex	0.102	−1.01	0.31
	*R*% *f*_H_ᶧ	Longevity	0.016	−1.63	0.10
	*R*% *f*_H_ᶧ	GSI (females only)	1.26	1.74	0.08

*Notes*: For all cases, the top three independent variables were selected using random forest analysis and simplified according to the lowest AICc values. The cross (ᶧ) identifies tests where *f*_H_ was temperature corrected. Significance was tested at *α* = 0.05 for all cases. Bold indicates statistical significance (α=0.05).

### Non-behavioral factors driving variability in *f*_H_

One-way ANOVAs were used to compare routine *f*_H_, minimum *f*_H_, resting *f*_H_, maximum *f*_H_, scope for *f*_H_ (difference between maximum and resting *f*_H_), and the percent of routine *f*_H_ within the scope for *f*_H_ between the two sexes. Relationships between *f*_H_ and condition, secondary sexual characteristics, time in the spawning channel, and diel patterns (i.e., day versus night) were explored with linear mixed effect models with AR(1) correlation structure and individual fish as a random effect to account for collinearity and repeated measures (*nlme* package; Pinheiro et al. 2014) (Table 1). For all cases, random forest analysis (*random forest* package; Liaw and Wiener 2015) was used to select the top three covariates that explained most of the variation in the response variable (according to %IncMSE) to avoid overfitting the models. Linear mixed effect models were then simplified using stepwise model selection according to the lowest AICc values. Next, individual rank order repeatability in *f*_H_ during the day and during the night (day starting at 6:30; night starting at 20:00) was determined using Spearman’s rank correlation within each sex.

Given that *R*%*f*_H_ is a continuous variable bound between 0 and 1, a beta regression model (*betareg* package, Zeileis et al. 2016) was used to determine whether *R*%*f*_H_ was related to longevity on the spawning ground (determined by counting the number of days that the fish spent in the spawning channel). A second beta regression model was then used to determine whether *R*%*f*_H_ was related to secondary sexual characteristics (Kieschnick and McCullough, 2003). Once again, random forest was used to select the top three covariates that most explained the variability in the response variable (according to %IncMSE) to avoid overfitting the models. Beta regression models (*betareg* package; Zeileis et al. 2016) were then further simplified using the model variation with the lowest AICc value.

### Relating *f*_H_ to spawning behavior

The *f*_H_ associated with each spawning behavior was determined by taking the average *f*_H_ for 15 min around the observed behavior (5 min before, during and after). A linear mixed effect model with AR(1) correlation structure and a beta regression model tested whether the average *f*_H_ and *R*%*f*_H_ (for each 15 min interval) differed among the three behaviors (Table 2). Both models had individual as a random effect to account for repeated measures (*glmmTMB* package; Brooks et al. 2017 for beta regression with repeated measures). The linear mixed effect model was then repeated using within individual differences in average *f*_H_ among behaviors as the response variable to test whether differences were related to the type of behavioral shift (e.g., change in average *f*_H_ from subordinate to dominant versus change in average *f*_H_ from dominant to aggregation). In this case average *f*_H_ was not temperature detrended, since changes in average *f*_H_ were quantified for an individual and temperature hardly varied between behavioral shifts (0.02 ± 1.05°C). Instead, average temperature and differences in temperature among behavior shifts were included as covariates. All models were repeated to determine whether variation in average *f*_H_ could be further related to sex, secondary sexual characteristics, longevity, or density on the spawning ground (random forest analysis percent variance explained < −46% for all cases), and GSI in females. Models were then simplified according to the lowest AICc value. Finally, cox proportional hazard analysis (*survival* package [(Therneau, 2015)]) was used to determine the relationship between GSI and longevity in females (proportional-hazards assumption *P*-value > 0.05).

**Table 2:** Statistical analyses (three tests) comparing *f*_H_ to spawning behavior.

Model type	Response variable	Independent variables	df	*F*	*P*
Linear regression with individual as a random effect	*f*_H_ resids*ᶧ	Behavior	272	0.29	0.75
		Time	272	0.01	0.90
	Δ*f*_H_ between behaviors	Behavior transition type	67	0.76	0.58
					
			**Std. error**	***z***	***P***
					
Beta regression with individual as a random effect	*R*% *f*_H_*ᶧ	Aggregation	0.13	−2.47	**0.01**
		Dominant	0.11	−0.36	0.72
		Subordinate	0.10	−0.69	0.09

*Notes:* Significance was tested at *α* = 0.05 for all cases. Asterisk (*) represents mean heart rate values over 15 min intervals. The cross (ᶧ) identifies tests where *f*_H_ was temperature corrected. Change (Δ) in *f*_H_ between behaviors represent change (Δ) in *f*_H_ during transitions from one behavior to another (e.g., dominant to subordinate). Bold indicates statistical significance (α=0.05).

All statistical analyses were conducted in RStudio. Models were tested at a 95% confidence level (*α* = 0.05), and all model assumptions were validated by testing for normality and inspecting the distribution of residuals.

## Results

### *f*_H_ profile

**Fig. 1:**
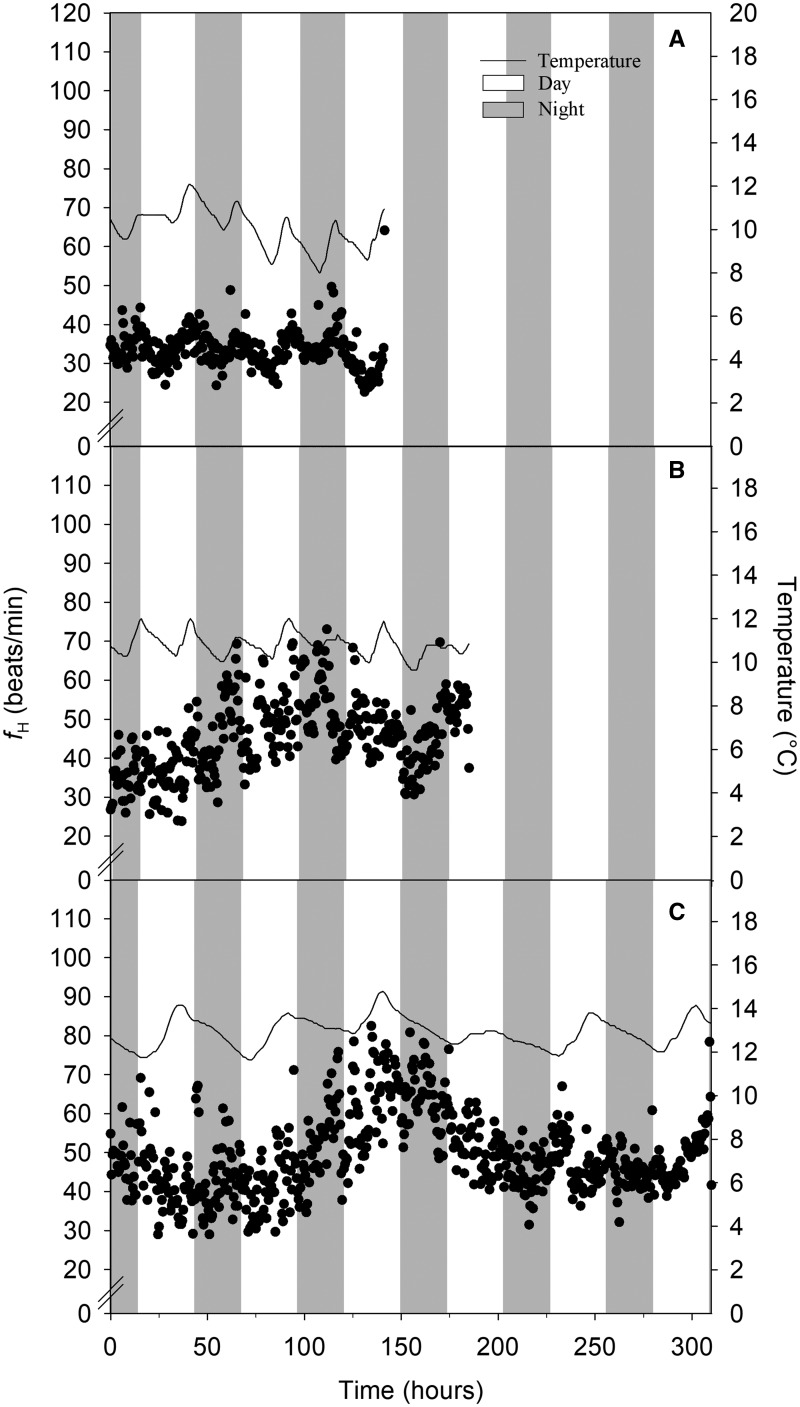
Scatterplot showing the heart rate (*f*_H_) (beats min^−1^) trace (raw data) of an individual spawning male (**A**), spawning female with GSI=9.8% that did not spawn (**B**), and spawning female with GSI=0% that did spawn (**C**) averaged for every half hour (black dots). *f*_H_ was recorded from spawning channel entry (after 1 day recovery) to mortality. Dashed line is the regression line for the water temperature. White bands represent daytime (6:30) and gray bands represent night (20:00).

**Fig. 2:**
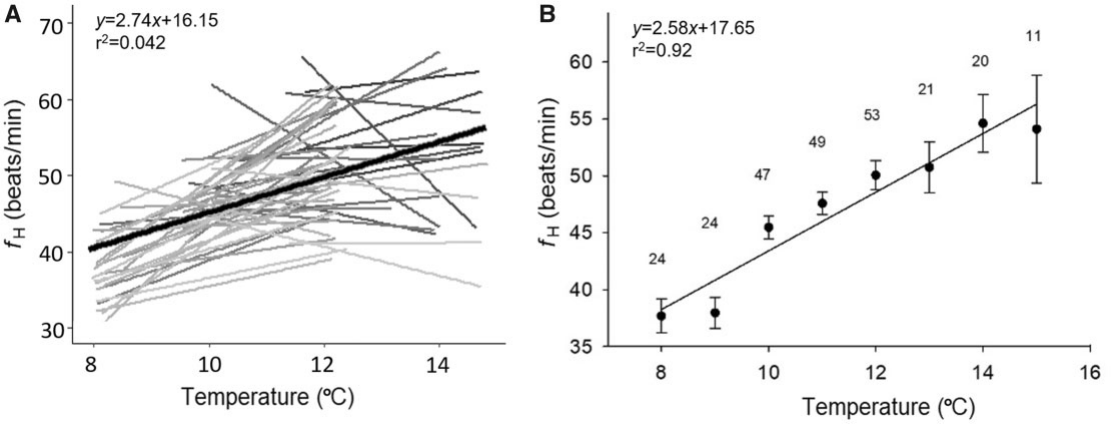
Regression plot showing **A**) individual slopes and **B**) mean *f*_H_ of 55 spawning sockeye salmon in response to water temperature increase in 1°C increments. The regression line shows that A) *f*_H_ increases according to the equation *f*_H_=2.74 (temperature, °C)+16.15, with a coefficient of determination (*r*^2^) of 0.042 (*P*-value<0.05).

For each individual, *f*_H_ fluctuated throughout the study period (Fig. 1). Overall maximum, minimum, resting, routine, and scope for *f*_H_ did not differ between sexes (all *P*-values > 0.05; values presented in Table 1). Furthermore, there were no differences in *f*_H_ parameters between sexes even after *f*_H_ was temperature corrected (*P*-value > 0.05 for all cases) and the daily average *f*_H_ (over 24 h) was not repeatable for either sex (*r*_s_* *< 0.25 for both). Temperature, however, had a positive effect on *f*_H_ (*r*^2^ = 0.92; *P*-value < 0.01; Fig. 2), where *f*_H_ increased by 2.7 ± 2.1% (mean ± SE) per 1°C increase.

**Fig. 3:**
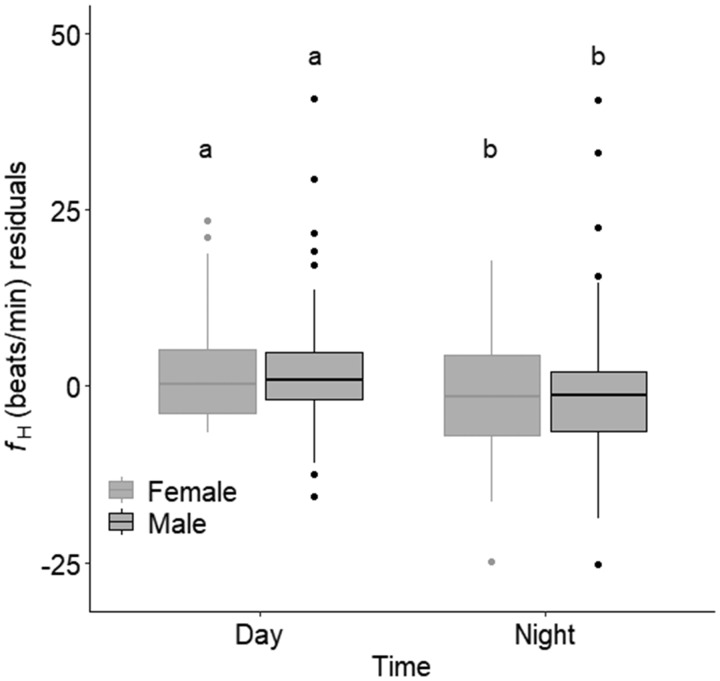
Boxplot showing the diurnal average, upper quartile, lower quartile, and standard error of spawning female (gray, *n* = 29) and male (black, *n* = 26) sockeye salmon percent routine *f*_H_ within scope for *f*_H_ (*R*%*f*_H_) over a spawning period. *f*_H_ data were temperature detrended to remove the effect of temperature on *f*_H_. Different letters show significant differences among groups (*P *<* *0.001).

**Fig. 4:**
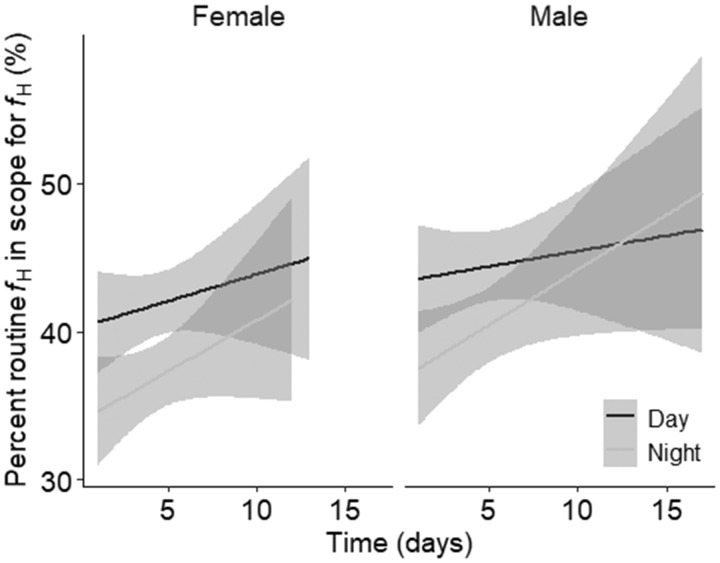
Linear mixed effect model showing the changes in diel heart rate (*f*_H_) as percent routine within scope for *f*_H_ (*R*%*f*_H_) over time in spawning male (left) and female (right) sockeye salmon. Gray line represents the *f*_H_ during the day, black line represents *f*_H_ during the night. Standard error is shown in gray.

**Fig. 5:**
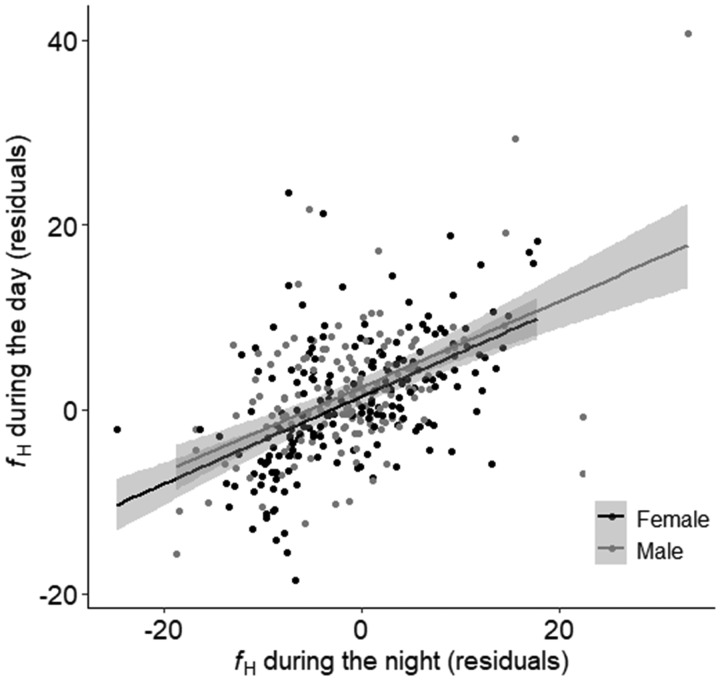
Linear mixed effect model showing the relationship between heart rate (*f*_H_) measured at night and *f*_H_ measured the subsequent day. Gray line represents the relationship within females, black line represents relationship within males. Gray dots represent individual females, black dots represent individual males. Standard error is shown in gray.

*R*%*f*_H_ (41.6 ± 1.3%) did not differ between sexes and neither did variability in *f*_H_ (determined by SE) differ between sexes (*P*-value > 0.05 for both; female SE = 0.093, male SE = 0.10). *R*%*f*_H_ did not affect either longevity or secondary sexual characteristics for either sex (all *P*-values > 0.05). However, for both sexes, *f*_H_ (temperature detrended) followed a diel pattern where *f*_H_ during the day was on average 2 ± 0.3% higher than *f*_H_ at night (Table 1 and Fig. 3). The linear mixed effects model relating *f*_H_ with time suggested that, despite the variability among individuals, *f*_H_ during the day and night increased over the spawning period (Table1 and Fig. 4). While these trends did not differ between sexes (removed from the model during model simplification), the diel trend appeared to weaken faster in males than in females (Fig. 4). The change in average *f*_H_ over time could not be explained by secondary sexual characteristics, longevity, or fish density on the spawning ground (all *P*-values > 0.05). However, irrespective of time since arrival to the spawning ground, an individual’s *f*_H_ at night was positively related to *f*_H_ of the subsequent day at the individual level (Table 1 and Fig. 5).

### Relationship between heart rate and behavior

**Fig. 6:**
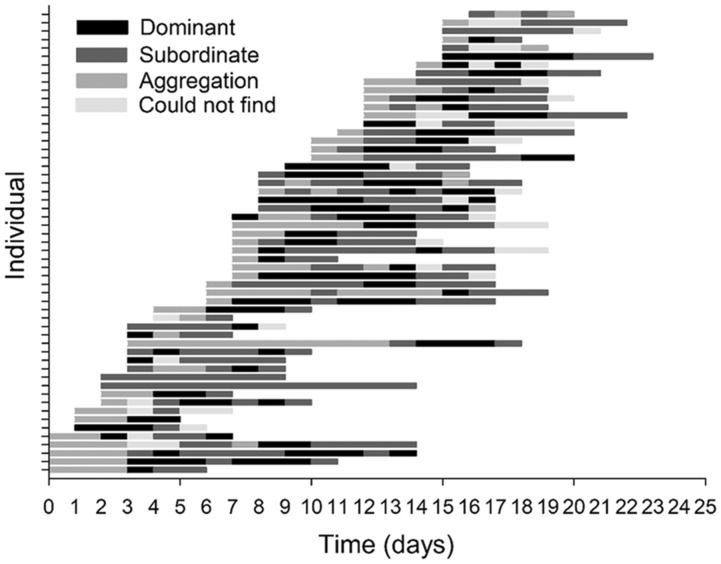
Observed time (day) spent on each spawning behavior status. Gray represents aggregation behavior status (fish were clustered and holding in pools), dark gray represents subordinate behavior status (fish were attempting to take over the territory of another fish, sneak onto a nest with a spawning female, or holding alone), and black represents dominance behavior (were in a position to spawn, meaning females were on a nest, digging and chasing other fish, and males were defending a territory and outcompeting other males when challenged). Light gray represents when the fish could not be found after several passes with two observers.

**Fig. 7:**
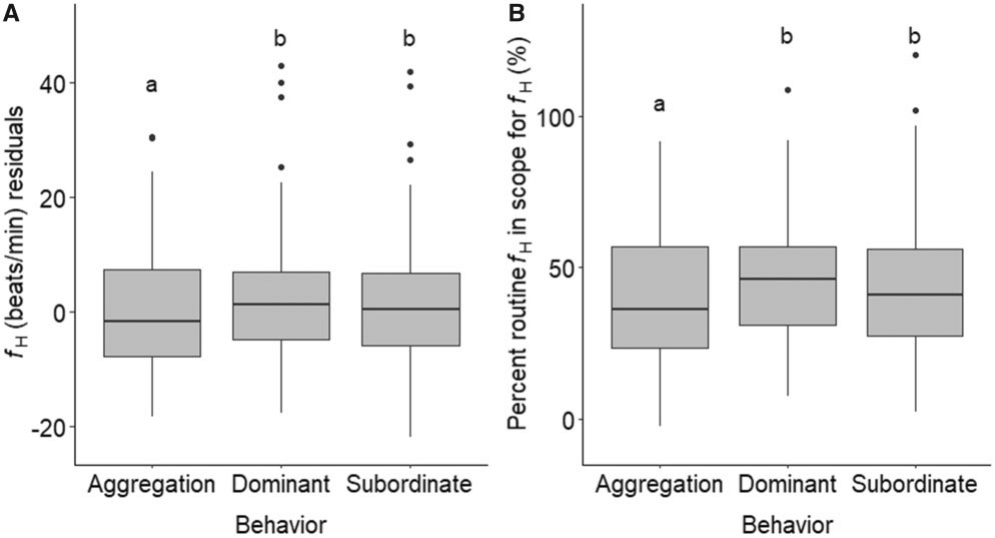
Mean heart rate (**A**) and routine *f*_H_ within scope for *f*_H_ (*R*%*f*_H_) (**B**) during the three behavioral states (dominant, subordinate, and aggregation). Letters represent significant differences in *f*_H_ among behaviors.

**Fig. 8:**
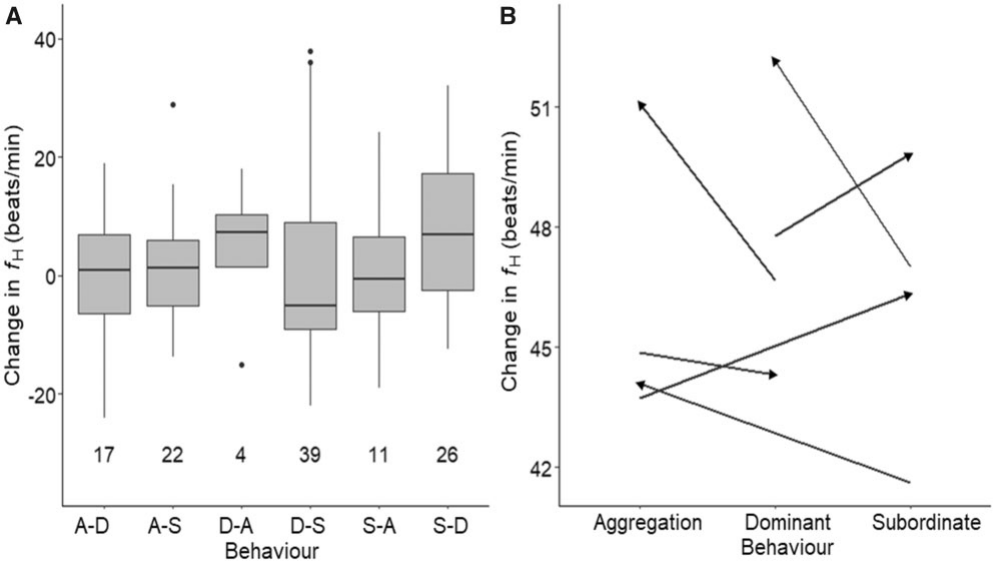
Individual variation in average changes in heart rate (*f*_H_) within scope for *f*_H_ (*R*%*f*_H_) (**A**) and magnitude of change in *f*_H_ (**B**) during shifts among the three behavioral states (dominant to aggregation [DA], subordinate to aggregation [S–A], aggregation to dominant [A–D], subordinate to dominant [S–D], aggregation to subordinate [A–S], and dominant and subordinate [D–S]). Individual was treated as a random effect to account for repeated measures. Number below boxplots show the sample size for each behavioral transition.

On average, fish were observed for 7.21 ± 0.4 days before they died. During this period, individuals were observed in aggregation for 1.69 ± 0.2 days, subordinate for 3.4 ± 0.3 days, and dominant for 2.16 ± 0.1 days (Fig. 6). Overall, *R*%*f*_H_ differed among the three behavioral states (Table 2), where *R*%*f*_H_ was approximately 6.2% and 1.2% lower during aggregation compared with during subordinate and dominant behaviors, respectively (Fig. 7). However, this was not apparent when visualizing the data and results could be an artifact of different sample sizes (*n*_aggregation_ = 68 vs. *n*_subordinate_ = 153 vs. *n*_dominant_ =111). Additionally, there was no effect of sex, secondary sexual characteristics, or fish density on average *f*_H_ during each behavioral state (all *P*-values > 0.05). Furthermore, there was no significant differences detected when individual changes in average *f*_H_ associated with transitions from one behavior to another were analyzed (Table 2). The largest change occurred when fish shifted from subordinate to dominant, where average *f*_H_ increased by 4.7 ± 18 beats min^−1^ (Fig. 8), but this was not significantly different from other shifts (Table 2). Over the spawning period, an individual was rarely dominant for more than one measurement cycle (Fig. 4). As the dominant fish retreated, another fish became dominant.

Individual changes in *f*_H_ could not be explained by the individual’s overall *f*_H_ parameters (i.e., *R*%*f*_H_, minimum *f*_H_, maximum *f*_H_, resting *f*_H_, or scope for *f*_H_; all *P*-values > 0.05—in models using random intercepts, random slopes, and random intercepts and slopes). Similarly, the changes in *f*_H_ when individuals shifted between behavioral states did not relate to secondary sexual characteristics or sex (all *P*-values > 0.05).

### Relationship between heart rate, behavior, and GSI (females only)

Neither *R*%*f*_H_ nor changes in average *f*_H_ with specific behaviors were related to an individual’s reproductive output, as measured by GSI, after death (*P*-values > 0.05 for both cases). Additionally, longevity on the spawning ground did not change with GSI (hazards ratio = 0.93, *z* = −0.97, *P*-value = 0.92), where on average both groups (females that spawned and females that failed to spawn) survived 7.40 ± 0.70 days on the spawning ground (males = 7.17 ± 0.20 days).

## Discussion

The present study aimed to test the hypothesis that *f*_H_ is related to reproductive investment (Franklin and Davie 1992; Ricklefs and Wikelski 2002; Sloman and Armstrong 2002; Perry et al. 2004; Clark et al. 2013) by exploring the relationship between *f*_H_ and reproductive status in spawning sockeye salmon. Our study did not detect any differences in *f*_H_ among the three different types of reproductive behaviors, sex, secondary sexual characteristics, spawning status, or fish density on the spawning ground. Thus, the present results do not support the hypothesis that individuals need to be capable of adjusting their *f*_H_ to attain a dominant status. This is the first study, to our knowledge, to explore the relationship between individual level *f*_H_ and behavior across several reproductive phases in spawning fish. However, our study is among multiple other studies failing to demonstrate that physiological performance is related to reproductive investment in fish (e.g., Wiegmann and Baylis 1995; Hatfield and Schluter 1999; Hanson et al. 2009), suggesting there is either no direct relationship between *f*_H_ and behavior, or that the sample design of the present study limited our ability to detect a trend.

### Characterization of spawning sockeye salmon heart rate

The *f*_H_ recorded in this study was comparable to the *f*_H_ recorded in previous studies using adult sockeye salmon (Table 3). Relatively small differences between the values obtained in this study and values in the literature could be due to different sockeye salmon populations used in the study (e.g., Quesnel vs. Early Stuart vs. Gates), where populations have unique physiological adaptations related to thermal environment and level of migratory difficulty (Eliason et al. 2011). Another reason for differences in recorded *f*_H_ could be due to the fact that, with the exception of Clark et al. (2009), previous studies that focused on spawning-phase fish restricted individuals to an enclosure (Sandblom et al. 2009; Clark et al. 2010), whereas fish in the present study were free-swimming throughout the spawning channel. Nevertheless, the present study did require surgical procedures and fish handling, which typically requires hours-to-days for full recovery (Raby et al. 2015; Prystay et al. 2017). Although fish were given 24 h to recover (restricted due to the nature of the project), the surgery may have still influenced reported *f*_H_ values. However even when fully exhausted (and our fish were not) the metabolic rate of a salmonid can fully recover in 24 h (Zhang et al. 2018).

**Table 3:** Adult sockeye salmon heart rate (*f*_H_) recorded in the present study and in previous literature

Population	Type	*f*_H_ (beats min^−1^)	Temperature (°C)	*n*	Study	Lifestage
Weaver	Routine	48	12	13	Sandblom et al. (2009)	Maturing
	Scope	60	10	11	Clark et al. (2009)	Mature
		(f_H_ ranged from 20 to 80 beats min^−1^)				
	Routine	∼40	11.5	11	Clark et al. (2010)	Maturing
		∼50	14			
Early stuart	Resting	70.1±2.3	15–20	9	Eliason et al. (2011, 2013)	Maturing
	Max	90.3±3.7				
	Scope for *f*_H_	∼25				
Quesnel	Resting	60.9±4.7	15–20	6	Eliason et al. (2011, 2013)	Maturing
	Max	97.7±7.2				
	Scope for *f*_H_	∼40				
Chilko	Resting	67.3±2.7	15–20	13	Eliason et al. (2011, 2013)	Maturing
	Max	94.0±3.2				
	Scope for *f*_H_	∼75				
Gates	Max	79.3±2.2	8–15	55	Present study	Mature
	Min	14.9±0.35				
	Resting	26.7±0.86				
	Routine	47.4±1.1				
	Scope for *f*_H_	52.6±2.2				

All data were obtained using Fraser River (British Columbia, Canada) sockeye salmon, where each population is unique (Eliason et al. 2011). Data were collected from migrating fish at different stages of migration/maturation, where *mature=*sockeye salmon have reached the spawning ground, and *maturing*=up-river migrating sockeye salmon.

For female sockeye salmon entering a spawning ground, *f*_H_ was previously shown to be 21% higher in females than males (Sandblom et al. 2009), potentially because female have larger gonads and a greater oxygen demand (Clark et al. 2009). Yet, spawning males spent 15% more time than females with their *f*_H_ >50 beats min^−1^ (although this result was non-significant) and routine oxygen consumption did not differ between sexes (Clark et al. 2009). Our study, in contrast, provided no support for either pattern: average *f*_H_ did not differ between sexes and the nighttime *R*%*f*_H_ was similarly 35–37% and the daytime *R*%*f*_H_ was similarly 40–43%. Also, *R*%*f*_H_ increased similarly with time in the spawning channels, but somewhat more so in the males that took longer (∼15 days) to spawn and die. Differences among studies could relate to difference in sampling period (<1 day previously vs. ∼7 days in the present study), individual sample size (11 and 13 previously vs. 55 in the present study), and *f*_H_ sampling frequency (e.g., continuous recording in Clark et al. 2009 vs. 6 s every 5 min in the present study). Regardless, mature female sockeye salmon do experience a higher mortality rate in response to stressors than males (Martins et al. 2012; Burnett et al. 2014) and have higher cortisol concentrations than males (Kubokawa et al. 1999; Sandblom et al. 2009; Hruska et al. 2010), both of which could elevate *f*_H_ (Farrell et al. 1988; cortisol is positively correlated with *f*_H_; Sandblom et al. 2009). Alternatively, previous research has shown that although the caloric breakdown of behaviors results in females burning on average 1109 more calories per day than males, the difference between sexes was not significant due to large intraspecific variation (Healey at el. 2003). Similarly, the variability in *f*_H_ may have masked differences in *f*_H_ between sexes in the present study (discussed further under the “Heart rate and spawning behaviors” section).

### Environmental factors influencing heart rate

After correcting for temperature, sockeye salmon *f*_H_ followed a diel pattern where *f*_H_ was higher during the day than at night, particularly at the start of the spawning period (Fig. 4). Diel variation in *f*_H_ has been documented in teleost species (Pickering and Pottinger 1983) including other studies conducted on spawning sockeye salmon, where diel patterns in *f*_H_ followed diel variation in visceral temperature (Clark et al. 2009, 2010). However, diel variation was not detected in previously recorded spawning sockeye salmon EMG records (Healey et al. 2003), suggesting behavior may not follow a diel pattern. Such variation in *f*_H_ is therefore likely driven by photoperiod, where time of day stimulates changes in other physiological parameters, such as hormones and blood plasma constituents, that were not included in the present study but can potentially drive changes in metabolic rate (Hoar et al. 1992; Farrell 1993; Vornanen 2017). Furthermore, the difference between day and night *f*_H_ decreased over time as the spawning period progressed (Fig. 4). A potential explanation for this trend is that circadian rhythms become more relaxed as fish approach senescence. Hruska et al. (2010) demonstrated that as sockeye salmon senesce, hormone and metabolite levels fluctuate, while lactate and cortisol increase, and [Na^+^], [Cl^−^], and osmolality decrease, thus supporting the notion that fish undergo physiological pressure as they approach senescence. The intrinsic link between the cardiovascular system and the endocrine system suggests that such changes in hormone and metabolites can further influence *f*_H_ ([e.g., *f*_H_ increases with increasing cortisol; Sandblom et al. 2009] discussed in Hoar et al. [1992] and Farrell [1993]; Vornanen 2017). Additionally, gross somatic energy reserves, including glucose levels, decrease over time (Rodnick and Gesser 2017), implying there is less available energy to maintain the frequent muscle contractions required for elevated *f*_H_ (Hruska et al. 2010). Therefore, it is possible that while overall maximal routine *f*_H_ (i.e., day) remains constant, *f*_H_ at night must increase to maintain sufficient cardiac output for survival (according to the equation that cardiac output = stroke volume × *f*_H_; Priede and Tytler 1977). Lastly, the slower rate of change in *f*_H_ observed in females (indicated by the shallower slope) may be due to differences in plasma hormones and metabolites compared with males (e.g., higher cortisol), and possibly because females have a higher hemoglobin concentration than males, increasing the female’s blood–oxygen carrying capacity (Clark et al. 2009).

In addition to temperature and diel variation, density of individuals would likely have an effect on behavior (Montero et al. 1999; Spence and Smith 2005; Tentelier et al. 2016). In the present study, fish density on the spawning ground was not correlated with *f*_H_ or behavior (see the “Heart rate and spawning behaviors” section). This is probably because the present study was conducted in an artificial spawning channel, where the number of fish entering the spawning channel was controlled, and fish were able to distribute themselves unevenly within the spawning channel reducing competition. This could also be due to the poor record of the actual fish density on the spawning channel, given that the electronic counter used to assess fish numbers was malfunctioning during the time of this study. Thus, we had to use estimates from the electronic counter data which could have been erroneous. Future research is required to further investigate the relationship between *f*_H_ and density of spawning sockeye salmon.

Lastly, predation pressure is another environmental factor that has been shown to affect *f*_H_ (Johnsson et al. 2001; Donaldson et al. 2010). However, this study was conducted in a controlled spawning channel, where redd predators were kept out. With the exception of the odd bear and bird predation, the sockeye salmon were generally protected from predators during spawning. Future studies are required to investigate whether salmon spawning in areas with more natural predator burdens would show the same *f*_H_ patterns.

### Heart rate and spawning behaviors

Time associated with each behavioral status during spawning varies among sockeye salmon populations (Healey et al. 2003). In the present study spawning sockeye salmon spent the most time as subordinate and the least amount of time in the aggregation phase (Fig. 6). Using EMG data to estimate the caloric consumption of locomotor activities during spawning, Healey et al. (2003) suggested that it was more energetically expensive to be dominant or subordinate rather than be engaged in an aggregation. A similar trend is obtained when using the equation for the relationship between *f*_H_ and metabolic oxygen consumption (MO_2_) from four Pacific sockeye salmon populations (Early Stuart, Chilko, Quesnel, and Nechako; data provided by Eliason and used in Eliason et al. 2011) to convert the *f*_H_ data from the present study to MO_2_, where the metabolic costs of each behavioral state can be roughly estimated as 2.7 ± 0.3 mg O_2_ min^−1 ^kg^−1^ (mean^ ^±^ ^SE), 2.6 ± 0.2 mg O_2_^ ^min^−1 ^kg^−1^, and 1.3 ± 0.2 mg O_2_^ ^min^−1 ^kg^−1^ for dominant, subordinate, and aggregation status, respectively. Other than the fact that the *f*_H_ to MO_2_ conversion equation was derived using different sockeye salmon populations with different physiological tolerances and performance levels (Eliason et al. 2011), potential discrepancies in these estimates are likely because, according to the Fick principle, *f*_H_ is only one of the components driving MO_2_ (Eliason et al. 2013; Farrell and Smith 2017). Other components include stroke volume (to determine cardiac output) and the arteriovenous oxygen extraction, which were not measured in the present study. Further discrepancies could also be caused by limited data, where delayed peak and *f*_H_ recovery post-exercise, extending beyond the 15-min intervals (Raby et al. 2015; Prystay et al. 2017).

The natural variability in *f*_H_ within and among individuals in the population may have masked differences in *f*_H_ among behavioral states. One possible explanation is that the changes in morphology (e.g., gonads) and hormones associated with senescence (Hruska et al. 2010; Rodnick and Gesser 2017), and varying environmental factors (e.g., temperature and diel variation, see the “Environmental factors influencing heart rate” section) are influencing individuals differently, causing variation in physiological (including *f*_H_) responses among individuals. Another possible explanation is that all fish studied had a broad scope for *f*_H_ since it has been shown that aerobic scope is positively related to whether a salmon can complete a spawning migration (Farrell et al. 2008). The true maximum *f*_H_ was not measured in the present study (to avoid interfering with spawning behaviors), potentially skewing the true variation in scope for *f*_H_ among individuals in the present study. Lastly, the lack of difference in *f*_H_ among behaviors could be due to the behavior data being too coarse. Healey et al. (2003) showed that holding on a redd was more energetically costly for females than males because they spent more time holding than males. This suggests that the duration spent on each behavior status strongly influences the metabolic cost. However, time spent at each behavior status was not recorded in the present study. Instead, behavior was only monitored once a day, and occasionally an individual was not found. As such, behavior immediately before and after the assessment were unknown, and behavior status could have shifted soon after the behavior was recorded. Future studies investigating the relationship between *f*_H_ and reproductive investment in spawning salmon should include accelerometer data for more refined behavior assessments, as well as measure maximum metabolic performance and changes in hormones associated with senescence.

Irrespective of time spent at each behavior status, we predicted that shifting between behavior status would result in a change in *f*_H_ at the individual level. Aggregation requires energy to hold position (Healey et al. 2003), however this behavior often occurs in pools (personal observation) where there is slower moving water, and in groups (Esteve 2005) where a fish can seek shelter in lower flow behind another fish. In contrast, subordinate and dominant behaviors require burst swimming, and thrashing in fast flowing water (Healey et al. 2003; Esteve 2005). However, we found no conclusive evidence that *f*_H_ differs among behaviors (Figs. 7 and 8). These results do not coincide with the Healey et al.’s (2003) EMG data, where caloric expenditures for both sexes were significantly larger during dominant and subordinate status compared with aggregation. Instead, increased metabolic demand associated with dominant and subordinate behaviors could have been met by changes in other cardiovascular parameters, such as stroke volume (Eliason et al. 2013; Farrell and Smith 2017). Additionally, lack of significant difference in *f*_H_ between behavior status could be because all behaviors involve holding, which is energetically expensive especially given that the fish are approaching senescence (Healey et al. 2003). Burst swimming associated with subordinate and dominant status occur less frequently than holding behaviors and could have been met by short bursts of anaerobic metabolism that resulted in non-detectible changes in *f*_H_ (Wood 1991). Coupling spawning salmon *f*_H_ data with accelerometry data in future studies would provide further insight on the fine-scale relationships between *f*_H_ and reproductive behaviors (e.g., Tsuda et al. 2006).

### Heart rate and longevity

Female longevity was independent of whether the female spawned, and independent of *R*%*f*_H_. Although an individual’s routine *f*_H_ changed over time, the rate of change in *f*_H_ was low for both day and night (slopes of ∼1% or less per day; Fig. 4). The lack of relationship between overall *f*_H_ and an individual’s longevity, reproduction behavior, and an individual’s spawning success or failure (although measured in females only) suggests that reproductive performance is likely driven by a different factor or, more likely, a complex combination of physiological variables (e.g., hormones, blood plasma constituents, or stored protein content; Hoar et al. 1992; Farrell 1993; Hendry and Berg 1999). It could also be argued that the activities of each reproductive behavior may not have been as energetically taxing as expected, resulting in no trade-off for change in *f*_H_, or that fish protect the heart function and performance throughout the 7 days of spawning. However, we also acknowledge that to avoid losing biologgers, this study was restricted to using fish that succeeded to migrate and reach the spawning channel, thereby potentially only including fish of similar condition and potentially excluding individuals with lower condition.

## Conclusion

Our study extends the findings from previous studies by exploring the relationship between *f*_H_ and reproductive behavior at the individual level. While the present study is the first, to our knowledge, to explore the relationship between individual level *f*_H_ and behavior across several reproductive phases in spawning fish, it adds to previous studies in fish that failed to establish a relationship between physiological performance and reproductive investment (e.g., Wiegmann and Baylis 1995; Hatfield and Schluter 1999; Hanson et al. 2009). The present study has revealed the complexity of the relationships between *f*_H_ and reproductive behaviors in wild spawning sockeye salmon and has demonstrated the importance of considering environmental factors when exploring among and within individual variation in future physiological and bioenergetic studies. Overall trends in *f*_H_ and behavior status were not seen, but we cannot eliminate the possibility that they were masked due to large interindividual variation in *f*_H_. Future studies should continue to investigate the relationship between physiological performance and reproductive investment in wild animals to enhance our current understanding of ecological processes in changing environments.

## Author contributions

S.J.C., E.JE., S.G.H., D.A.P., A.P.F., and T.S.P. conceived the ideas and designed the methodology. T.S.P., R.d.B., and K.S.P. collected the data. T.S.P. analyzed the data and led the writing of the manuscript. All authors contributed critically to the drafts and gave final approval for publication of the manuscript.
